# Where Am I? Niche constraints due to morphological specialization in two Tanganyikan cichlid fish species

**DOI:** 10.1002/ece3.6629

**Published:** 2020-08-12

**Authors:** Lukas Widmer, Adrian Indermaur, Bernd Egger, Walter Salzburger

**Affiliations:** ^1^ Department of Environmental Sciences Zoological Institute University of Basel Basel Switzerland

**Keywords:** adaptive radiation, Cichlidae, Lake Tanganyika, phenotypic plasticity, reciprocal transplant experiment

## Abstract

Food resource specialization within novel environments is considered a common axis of diversification in adaptive radiations. Feeding specializations are often coupled with striking morphological adaptations and exemplify the relation between morphology and diet (phenotype–environment correlations), as seen in, for example, Darwin finches, Hawaiian spiders, and the cichlid fish radiations in East African lakes. The cichlids' potential to rapidly exploit and occupy a variety of different habitats has previously been attributed to the variability and adaptability of their trophic structures including the pharyngeal jaw apparatus. Here we report a reciprocal transplant experiment designed to explore the adaptability of the trophic structures in highly specialized cichlid fish species. More specifically, we forced two common but ecologically distinct cichlid species from Lake Tanganyika, *Tropheus moorii* (rock‐dweller), and *Xenotilapia boulengeri* (sand‐dweller), to live on their preferred as well as on an unpreferred habitat (sand and rock, respectively). We measured their overall performance on the different habitat types and explored whether adaptive phenotypic plasticity is involved in adaptation. We found that, while habitat had no effect on the performance of *X. boulengeri*, *T. moorii* performed significantly better in its preferred habitat. Despite an experimental duration of several months, we did not find a shift in the morphology of the lower pharyngeal jaw bone that would be indicative of adaptive phenotypic plasticity in this trait.

## Graphical abstract

We performed a reciprocal transplant experiment in which we forced two highly specialized cichlid fish species from African Lake Tanganyika to live on their preferred as well as on an unpreferred habitat. We found that one of these species (the rock‐dweller *Tropheus moorii*) performed significantly better in its preferred habitat. We did not find evidence for adaptive phenotypic plasticity in trophic structures in these species.

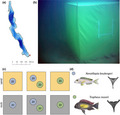

## 1 INTRODUCTION

Adaptive radiation is the rapid ecological and morphological diversification of an ancestral—often generalist—species into an array of specialized descendants (Schluter, 2000). Such a burst of diversification can occur (a) after the emergence of a novel trait (in this context termed “key innovation”) allowing the exploitation of previously inaccessible niches; (b) following the colonization of an empty or underutilized environmental space providing ecological opportunity; or (c) in the aftermath of extinction events carrying off antagonists and thus liberating previously occupied niches (Schluter, 2000). A common axis of morphological diversification during adaptive radiation is related to food resource specialization, as exemplified by the correlation between food type and beak shape in Darwin's finches (Snow & Grant, 2006), between the hunting ground and the coloration of Hawaiian spiders (Gillespie, Benjamin, Brewer, Rivera, & Roderick, 2018; Roderick & Gillespie, 1998), and between diet and trophic morphology in East African cichlid fishes (Muschick, Indermaur, & Salzburger, 2012).

The correlation between the phenotype of a species and the environment that this particular species inhabits is—according to Schluter (2000)—one out of four criteria to detect an adaptive radiation, the others being common ancestry, rapid diversification, and “trait utility.” The latter criterion refers to the performance of a trait in a given environment and, hence, directly relates to the “adaptive” nature of a radiation. The expectation here is that a particular trait value or phenotype performs best in a given environment and/or with respect to a particular food source (that is, the ecological niche) to which it is adapted to. While phenotype–environment correlations have been established for all main examples of adaptive radiation (reviewed in Schluter, 2000), experiments testing the performance of phenotypes in natural settings are rare.

The assemblages of cichlid fishes in the African Great Lakes Victoria, Malawi, and Tanganyika are textbook examples of adaptive radiations (Salzburger, 2018; Salzburger, Bocxlaer, & Cohen, 2014; Sturmbauer, Husemann, & Danley, 2011). African lake cichlids exhibit a great variety of ecological specializations including, among others, sand filtering, scale‐eating, invertebrate picking, algae grazing, mollusk cracking, and preying fish (Fryer & Iles, 1972; Konings, 2015). On the other hand, many specialized cichlid species are inherently opportunistic and deviate from their usual diet if presented with a new, more easily accessible food source (Ribbink, 1990).

The cichlids' potential to rapidly exploit and occupy a variety of different niches has previously been attributed to the particular anatomy of their trophic structures including the presence of a pharyngeal jaw apparatus (Liem, 1973). This second set of jaws in the pharynx of cichlids is primarily used for food processing, allowing a functional decoupling between food uptake and mastication (Liem, 1973). While modified pharyngeal jaw structures are found in other groups of fish as well, the ones of the cichlids are characterized by a sutured lower pharyngeal jaw bone (LPJ) (note that this trait of a unified LPJ is shared with closely related percoid families such as labrids or embiotocids (Liem & Greenwood, 1981; Galis & Drucker, 1996; Hulsey et al., 2006)). Cichlids show a great diversity in the morphology of the pharyngeal jaw apparatus, and it has been demonstrated that LPJ shape and dentition are correlated to the feeding ecology of a species (Liem, 1973; Meyer, 1989; Muschick et al., 2012). Through common garden experiments involving different feeding regimes, it has further been shown that in some cichlid species, the morphology of the LPJ can be experimentally altered (Gunter et al., 2013; Huysseune, 1995; Meyer, 1989; Muschick, Barluenga, Salzburger, & Meyer, 2011). This suggests that LPJ diversification may initially be facilitated by adaptive phenotypic plasticity, that is, the capacity of a genotype to produce more suited phenotypes corresponding and adapting to novel environmental conditions (Schneider & Meyer, 2017; West‐Eberhard, 2005).

**FIGURE 1:**
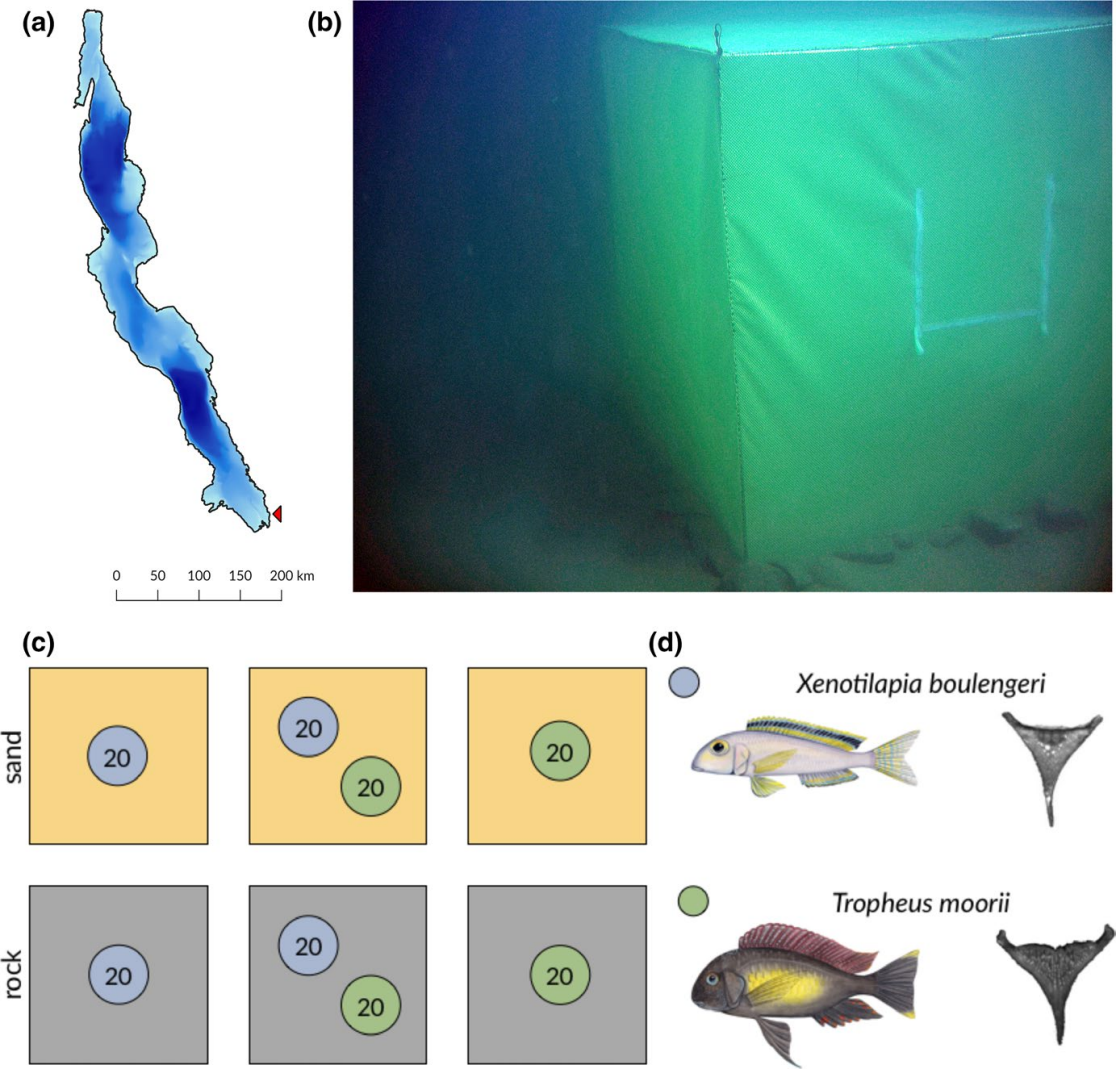
(a) Map of Lake Tanganyika with the study location at Kalambo Falls Lodge indicated by a red arrowhead. (b) Underwater image of one of the six cages used in the experiment (the entrance is visible on the front part; photograph A. Indermaur). (c) Experimental setup of reciprocal transplant experiment in seminatural conditions. Squares represent cages, the coloring indicates substrate type (sand: yellow, rock: gray), and the circles show the number of individuals used in each cage per species (blue: *Xenotilapia boulengeri*; green: *Tropheus moorii*). (d) Illustrations of the species used in this experiment (Illustrations by J. Johnson) and exemplary pictures of lower pharyngeal jaw bones for each species

In this study, we report a reciprocal transplant experiment under semi‐natural conditions within Lake Tanganyika to test for local adaptation with respect to benthic habitat types and to examine the possible contribution of phenotypic plasticity to medium‐term adaptation in two common but ecologically and behaviorally very distinct endemic cichlid species, *Tropheus moorii* (Boulenger, 1898) and *Xenotilapia boulengeri* (Poll, 1942). *Tropheus moorii* is abundant in the shallow rocky habitats of southern Lake Tanganyika (down to a maximum depth of around 20 m), where it feeds on aufwuchs (algal flora growing on rocky substrate); this species exhibits a papilliform LPJ suited for grinding algae, fitting its herbivorous diet (Figure 1); it is highly territorial and vigorously defends its feeding territory against intrusion of other herbivores (Konings, 2015). *Xenotilapia boulengeri* occurs over sandy substrate throughout Lake Tanganyika; this species usually roams in large foraging groups and feeds on invertebrates which it filters from the upper layers of the sandy sediment (Konings, 2015); its LPJ is molariform and well suited to crush the small invertebrates that it extracts from the sand (Figure 1d). Data from a recent census study (Widmer, Heule, Colombo, Rueegg, Indermaur, Ronco & Salzburger, 2019) confirm that the two species show very little habitat niche overlap (Figure S1).

We used underwater cages installed over the benthic habitat of Lake Tanganyika to force experimental groups of both species—as well as mixed‐species groups to evaluate the effect of competition—to live for several months in two distinct habitat types (sand and rock), thus exposing each species to their native as well as to a foreign habitat. We then determined survival rates as well as individual growth rates as proxy for fitness. We predicted that resource specialization of the two investigated cichlid species will be reflected in lower survival and individual growth rates when being forced to live on foreign substrate and that additional competition, exercised through the presence of the locally adapted species, will intensify this effect. Additionally, to explore the possible contribution of phenotypic plasticity to local adaptation, we compared LPJ and body shape between experimental groups and a reference sample from the wild by means of geometric morphometric analyses. Both LPJ and body morphology/shape have been shown to be associated with feeding and habitat niche, respectively, in Lake Tanganyika cichlids (Muschick et al., 2012). As a consequence, we expected LPJ and body shape changes compared to the reference samples in experimental groups forced to live in foreign habitats.

## 2 MATERIALS AND METHODS

### 2.1 Study site and experimental design

The field experiments for this study were conducted in the bay off Kalambo Falls Lodge (8°37′23″S, 31°12′1″E) at the southeastern shoreline of Lake Tanganyika near Mpulungu, Republic of Zambia (Figure 1a), during two consecutive field seasons in 2011 and 2012. We used an installation consisting of six underwater cages (width = 2 m, length = 2 m, height = 2 m) positioned around 30 m offshore at a depth of 6–9 m in close proximity to each other. The cages were made of hollow steel frames covered on the sides and on top, but not at the bottom, by a sturdy net with a 6 mm mesh‐size (Figure 1b) (see Indermaur, Theis, Egger, & Salzburger, 2018). Three cages were set to emulate the rocky habitat in this area of Lake Tanganyika by completely covering the substrate with rocks collected from the immediate surroundings (in the following referred to as substrate type “rock”). In the other three cages, all rocks were removed leaving behind only homogeneous sandy substrate (referred to as “sand”) (Figure 1c). The cages were left for several days before starting the experiments, in order to establish natural conditions with respect to the substrate.

Two consecutive replicates of the reciprocal transplant experiment were performed, the first one lasting for 120 days and the second one for 160 days (due to travel arrangements we could not run the experiments for the exact same duration). Two common Tanganyikan cichlid species with opposing habitat preferences were used in our experiments: *T. moorii*, an aufwuchs feeder, which predominantly inhabits rocky areas, and *X. boulengeri*, which is a substrate filter feeder mostly found roaming and feeding over sand banks (Konings, 2015). The specimens used in the experiments were caught at the same location by SCUBA divers using fine mesh gill and hand nets (mesh‐size 6 mm) and targeting subadult individuals. In each round of the experiment, one “sand” and one “rock” cage were each stocked with 20 individuals of *T. moorii* and one “sand” and one “rock” cage were each stocked with 20 individuals of *X. boulengeri*. Thereby, one experimental group per species was allowed to stay on the native habitat (“home”) as a control group, while another group was forced to live on the foreign habitat (“away”) as treatment group. One “sand” and one “rock” cage per experimental round were stocked with a mixed experimental group consisting of 20 individuals each from both species (note that number of individuals used in this experiment resembles maximal densities observed in nature (Sturmbauer & Dallinger, 1994)). Experimental groups were randomly allocated to one of the cages to account for position effects. During the stocking of the cages, some specimens died, most likely due to compression complications, and had to be replaced the following day until the original stock number was reached (see Figure 1c). Prior to stocking them in the experimental cages in a 1:1 sex ratio, all specimens were sexed, measured (SL—standard length, TL—total length), weighted, photographed for 2D morphometrics, and fin clips were taken and stored in 96% ethanol as DNA samples for later identification; this procedure was performed under temporary anesthesia with clove oil (2–3 drops clove oil per liter water). During the entire experiment, experimental fishes were left unattended in the cages until they were recaptured by SCUBA divers using hand nets at the end of each experimental round. The same measuring and sampling procedure as described above was applied to all recaptured specimens, with the addition of dissecting the lower pharyngeal jaw bones (LPJs) for morphometric analysis. Fin clips and LPJs were then transported to the Zoological Institute of the University of Basel for further analysis.

### 2.2 Specimen matching

In order to match individual samples and, hence, the measurements from before and after the experimental treatment, we genotyped each specimen at four microsatellite loci following an established protocol (Theis, Ronco, Indermaur, Salzburger, & Egger, 2014). In brief, we extracted genomic DNA from fin clips using a MagNA Pure LC (Roche) with the DNA Isolation Kit II (Tissue) in the case of *T. moorii* and a 5% Chelex solution‐based extraction protocol in the case of *X. boulengeri* samples (Casquet, Thebaud, & Gillespie, 2012). All four microsatellite loci were amplified in a single multiplex PCR using published primers (Ppun5, Ppun7, Ppun21 (Taylor et al., 2002), Pzeb3 for *T. moorii* only (Van Oppen et al., 1997), UNH130 for *X. boulengeri* only (Kocher, Lee, Sobolewska, Penman, & McAndrew, 1998)). We used the QIAGEN^®^ Multiplex PCR Kit and 1 μL of DNA extract under the following PCR conditions: 95°C for 15 min and 40 3‐step cycles of 94°C for 30 s, 57°C for 90 s and 72°C for 60 followed by the end step at 60°C for 30 min. 1 μl of PCR product was resuspended in HiDi Formamide and, after the addition of a size standard (ABI LIZ(‐250)), analyzed on a 3130*xl* Genetic Analyzer (Applied Biosystems). Microsatellite peaks (allele sizes) were evaluated using Peak Scanner™ v1.0 (Thermo Fisher Scientific) and rounded with Tandem (Matschiner & Salzburger, 2009). The rounded values were used for sample matching with the R package Allelematch version 2.5 (Galpern, Manseau, Hettinga, Smith, & Wilson, 2012), allowing us to trace individual fish and to obtain individual‐level data for growth in length and weight.

### 2.3 Morphometrics

For geometric morphometric analyses of body shape, we used 522 photographs (taken before and after experimental runs), including 65 photographs from wild‐caught specimens as reference for body shape. Analysis of LPJ shape was based on 112 LPJs (including LPJs of 27 wild‐caught adults as reference) from the two replicates of the seminatural reciprocal transplant experiment (37 *T. moorii*, 75 *X. boulengeri*). We used a desktop office scanner (EPSON perfection V30/V300, resolution: 4,800 dpi) to obtain digital images of the cleaned jaws. Each scan included a ruler as size reference.

Following the procedure described in Muschick et al. (2012), “x” and “y” coordinates of 17 homologous landmarks for body shape and eight homologous landmarks and of 20 semilandmarks for LPJ shape were acquired in tpsDig2 (Rohlf, 2018). Landmark sliding was performed in R using the package *geomorph* version 3.1.0 (Adams & Otárola‐Castillo, 2013). From the initial 28 LPJ landmarks, we retained seven true landmarks and six semilandmarks, totaling in a set of 13 landmarks for morphometric analysis. Statistical analyses of the morphometric data of body shape as well as LPJ shape were performed in MorphoJ version 1.06d (Klingenberg, 2011).

### 2.4 Statistical analysis

To evaluate survival rate in the cage experiment between species and experimental conditions, we applied generalized linear mixed effect models (GLMM), with the dependent variable survival (0 = died and 1 = survived) and the fixed predictors initial weight, substrate (rock, sand), competition (single species cage or dual species cage), their interaction (substrate: competition), and sex (male, female). We included two variables as random effects: “replicate” in order to account for the relatively low number of replicates in the study design and “cages” to include any possible variability among the six cages.

For each survivor, we calculated absolute growth rates in g/day as well as specific growth rates, SGR = 100 * (ln (final weight) − ln (initial weight))/days) as described previously (Rajkov, Weber, Salzburger, & Egger, 2018). We regressed SGR with initial weight to correct for potential differences. Following Scharsack, Kalbe, Harrod, and Rauch (2007), the residual SGR (rSGR) values were used as a measure of relative growth performance. We evaluated relative growth between the different species and experimental conditions using linear mixed effect models, with the dependent variable rSGR and the fixed predictors substrate (rock, sand), competition (single species cage or dual species cage), and sex (male, female). Replicate and cage were included as random effects. All statistical analyses were performed in R version 3.5.1 (R Development Core Team, 2016).

## 3 RESULTS

### 3.1 Survival

At the end of the first replicate, we retrieved 72 of the 81 initially stocked specimens of *T. moorii* and 32 of the 104 (including replacements) stocked *X. boulengeri*. After the second round, we retrieved 65 of the 81 stocked *T. moorii* and 29 of the 81 stocked *X. boulengeri* (see Widmer, Indermaur, Egger, & Salzburger, 2020 for data). Across the experimental rounds and cages, the survival rate was much higher in *T. moorii* (84.5%) as compared to *X. boulengeri* (33.1%) (GLMM, *N* = 324, *z*_Species_ = −8.438, *p*_Species_ > .000). Survival in *T. moorii* was not affected by any of our predictor variables. In *X. boulengeri*, we found a significant negative effect of competition and of the interaction between substrate and competition on survival rate (GLMM, *N* = 162, *z*_Competition_ = 3.330, *p*_Competition_ = .001 and *z*_Substrate:Competition_ = −2.219, *p*_Substrate:Competition_ = .03).

### 3.2 Growth

**FIGURE 2:**
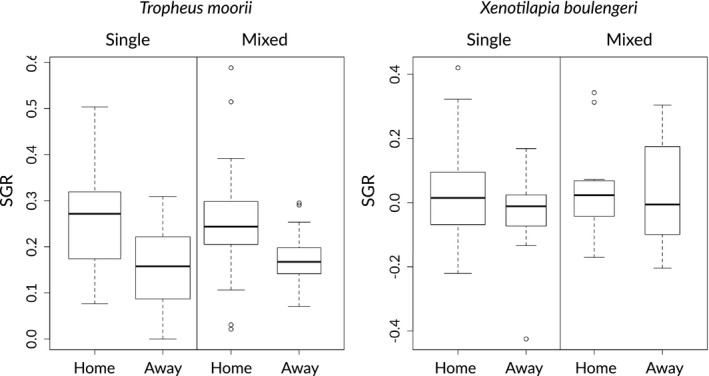
Specific growth rate (SGR) for *Tropheus moorii* and *Xenotilapia boulengeri* for different substrate (home = preferred habitat, away = unpreferred habitat) and competition conditions (single or mixed cages)

After genotyping the experimental fish (from before and after each round) at four microsatellite loci, 127 specimens of *T. moorii* and 55 specimens of *X. boulengeri* could be successfully matched using Allelematch (Galpern et al., 2012). These specimens were then used for growth rate analyses. The mean initial standard length of these specimens was 6.0 ± 0.9 cm for *T. moorii* and 8.0 ± 1.3 cm for *X. boulengeri* (Widmer et al., 2020). SGR was higher in *T. moorii* than in *X. boulengeri* across both experimental rounds and habitat types (ANOVA, *F* = 3,612, *p* < .001). For rSGR, there was no detectable difference between the two species across both experimental rounds and habitat types. We found that *T. moorii* had a significantly higher SGR (*F* = 13.07, *p* < .001) and rSGR (*F* = 27.2, *p* < .001) on their native substrate (“rock”) compared to the foreign one (“sand”) (Table 1; Figure 2). No such difference in SGR or rSGR was detected for *X. boulengeri*.

**TABLE 1:** Variance table of mixed effect models on specific growth rate (SGR) and relative growth performance (rSGR)

Whole dataset SGR				
Effect	num.*df*	den.*df*	*F*	*p*
Species	1	175.3	3,729.7	<.001^T^
Sex	2	175.4	1.24	.292
Substrate	1	166.0	6.86	.009^R^
Competition	1	166.3	0.00	.985

Effect	*Xenotilapia boulengeri* only				*Tropheus moorii* only			
	num.*df*	den.*df*	*F*	*p*	num.*df*	den.*df*	*F*	*p*
Sex	2	43.3	1.85	.169	2	121.4	2.19	.116
Substrate	1	49.1	2.51	.119	1	121.0	13.07	**<.001^R^**
Competition	1	49.4	1.30	.259	1	121.0	0.45	.506

Whole dataset rSGR				
Effect	num.*df*	den.*df*	*F*	*p*
Species	1	174.9	1.23	.268
Sex	3	174.5	0.88	.453
Substrate	1	174.1	23.34	**<.001^R^**
Competition	1	174.0	0.00	.951

Effect	*Xenotilapia boulengeri* only				*Tropheus moorii* only			
	num.*df*	den.*df*	*F*	*p*	num.*df*	den.*df*	*F*	*p*
Sex	2	47.5	2.37	.104	2	121.3	0.99	.374
Substrate	1	49.5	1.69	.199	1	121.0	27.2	**<.001^R^**
Competition	1	49.4	1.54	.220	1	121.0	0.02	.881

Note

*F*‐statistics were corrected with the Kenward–Roger approximation for mixed linear models. Significant effects (*p* < .05) are highlighted in bold and marked for the direction: *Tropheus moorii* (^T^), Substrate type “rock” (^R^).

### 3.3 LPJ and body shape

**FIGURE 3:**
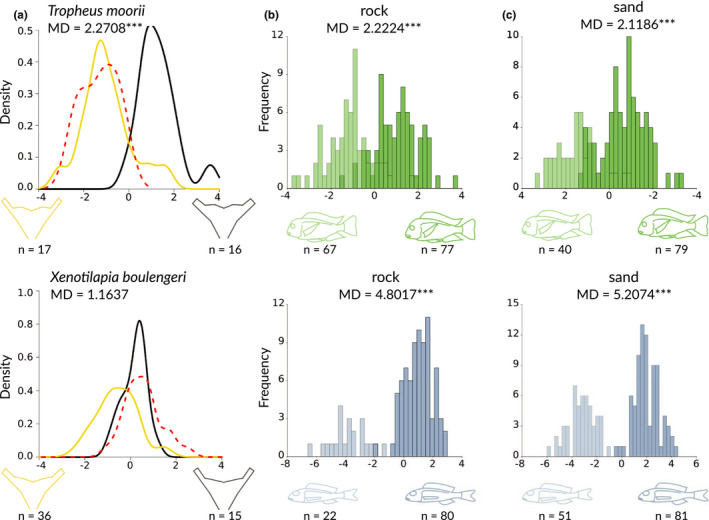
(a) Density plots of canonical variance (CV) scores for lower pharyngeal jaw shape in *Tropheus moorii* (upper panel) and *Xenotilapia boulengeri* (lower panel) by substrate type (sand = yellow, rock = black). The density function of wild‐caught specimens used as reference is shown as red dashed line. b) Frequency plots of CV scores for body shape in specimens at the start of each experimental round over the substrate type “rock” (dark shading) and at the end (light shading) for *T. moorii* (green coloration) and *X. boulengeri* (blue coloration). c) Frequency plots of CV scores for body shape in specimens at the start of each experimental round over the substrate type “sand” (dark shading) and at the end (light shading) for *T. moorii* (green coloration) and *X. boulengeri* (blue coloration). Outlines representing the maximal shape changes are displayed below each plot. n – sample size for each group; MD – Mahalanobis distance between experimental groups (habitat type “rock” vs. “sand”); *** – significant differences between Mahalanobis distances at *p* > .001

The landmark‐based principal component analysis (PCA) of the 111 LPJ (one outlier removed) showed a clear separation between the two species in the first two axes, which explained 74.9% of the total variation (Figure S2). In subsequent within‐species canonical variance analysis (CVA), shape differences in LPJ shape were detected in CV1 for the different substrate types in *T. moorii* (Mahalanobis distances (MD) = 2.2708, *p* < .001) but not in *X. boulengeri* (MD = 1.1637, *p* = .18) (Figure 3a). The PCA examining body shape over all specimens captured the marked morphological difference between the two species, as displayed by the high level (85.6%) of explaining variance in the first two PC axes. The CVA revealed significant morphological differences (deepening of body for *T. moorii* and slimming of body for *X. boulengeri*) along CV1 for both species in body shape during the course of the experiment on the different substrate types (*T. moorii*_rock_ MD = 2.2224, *p* < .001; *T. moorii*_sand_ MD = 2.1186, *p* < .001; *X. boulengeri*_rock_ MD = 4.8017, *p* < .001; *X. boulengeri*_sand_ MD = 5.2074, *p* < .001) (Figure 3b,c).

## 4 DISCUSSION

Here we report the results from a reciprocal transplant experiment involving two ecologically divergent benthic species of cichlid fishes from African Lake Tanganyika, the algae grazer *T. moorii*, which occurs exclusively in rocky habitats, and the invertebrate feeder *X. boulengeri*, primarily found over sandy substrate (Figure 1). The experiment was conducted under semi‐natural conditions in underwater cages in Lake Tanganyika and designed so that experimental populations were allowed to live on their preferred habitat or were forced to unpreferred substrate types (with and without competition in the form of mixed groups between the species) to test for local adaptation and the possible role of adaptive phenotypic plasticity in medium‐term adaptation to a novel environment.

In the two rounds of reciprocal transplant experiments, each lasting for several months, we observed relatively high mortality rates in *X. boulengeri* (especially over the unpreferred substrate type “rock”), while *T. moorii* exhibited high survival rates irrespective of substrate types. The presence of *X. boulengeri* did not affect survival rates in *T. moorii*, which is in line with the observation that *T. moorii* exhibit little territorial behavior toward members of other trophic guilds (Konings, 2015). The overall much lower survival rate in *X. boulengeri* may be attributed to the fragility of this species, as exemplified by the high mortality of *X. boulengeri* during handling and stocking of cages. If handling might differently affect the study species, our results on species‐specific survival rates in the different habitat types should be taken with caution.

With respect to growth rates, we could confirm the expected pattern that experimental populations should perform better in their native environment only for one of the species, *T. moorii* (Figure 2). The relatively high growth rates in *T. moorii* in comparison with *X. boulengeri*—also in the experimental groups over the unpreferred substrate (“sand”)—might be confounded by an unforeseen food source, algal growth on the mesh of the cages, which was observed to be scraped off by *T. moorii* specimens. In *X. boulengeri*, growth rates were low in any of the experimental groups. As *X. boulengeri* is known to roam over large swathes of substrate in search of food, the 4 m^2^ of substrate in the enclosures might not provide enough invertebrates for this species to feed upon, and it appears that no alternative food source was exploited by this species in the experimental cages.

The morphometric analyses of LPJ shape revealed that the LPJ of *X. boulengeri* raised over rock were not significantly different from the ones raised over sand (Figure 3a). In *T. moorii*, on the other hand, we found a significant difference in Mahalanobis distance (MD) between the sand versus rock experimental groups (Figure 3a). However, the comparison to wild‐caught *T. moorii* individuals from the same location revealed that these reference LPJs were more similar to the experimental groups from the foreign habitat (sand) than to the native one (rock), which is against our expectations. This pattern might be an artifact of the small number of reference specimens used for the comparison. An alternative explanation is the absence of other algae‐eating species in the experimental cages (e.g., species of the genera *Eretmodus, Petrochromis,* and *Simochromis*), which—under natural conditions—co‐occur and compete with *T. moorii*. The lack of competition in the cages might have led to an increased food (aufwuchs) availability and diversity for *T. moorii*, mediating the observed shift in LPJ morphology.

The otherwise little or inconclusive evidence for a plastic response in LPJ over the course of our experiments could have several reasons. It is possible, for example, that phenotypic plasticity is predominantly found and expressed more strongly in generalist species (Svanbäck & Schluter, 2012). Specialists, such as the species included in this study, might have lost this ability during the course of the radiation (Schneider & Meyer, 2017).Furthermore, both species feature oral jaws and teeth that are specialized to forage algae (*T. moorii*) and invertebrates (*X. boulengeri*), respectively. Such a degree of specialization in the oral jaws might prevent a shift toward alternative food sources. Finally, our study was conducted with wild‐caught specimens of unknown age. Although we were targeting subadults, it is possible that—at this life stage—phenotypic changes in LPJ morphology can no longer be induced via dietary shifts. In previous experiments, in which diet‐induced changes in LPJ morphology were observed, experimental fish were raised on an altered diet from the fry stage onwards (Muschick et al., 2011).

The observed changes in body shape (Figure 3b,c) are likely an effect of captivity and not of any experimental treatment, as all the shape changes occurred in a similar direction in all treatments (i.e., toward a slimming of the body). This might be because of the reduced swimming ranges induced by the experimental set up, reducing overall body muscle mass. In line with this, the effect was more pronounced in *X. boulengeri*, which, under natural conditions and compared to *T. moorii*, covers much larger distances swimming in foraging schools. Alternatively, the observed reduction in relative body height could be due to nutrition deficiencies and/or starvation of the encaged specimens, in particular in *X. boulengeri* (recall that *T. moori,* at least occasionally, scraped algae from the cages).

Taken together, the observed patterns in survival and growth in our experiment corroborate the high specialization of the study species to a particular habitat type and illustrate that specialist cichlids do not easily adapt to different environmental settings or exploit alternative food sources. Thus, the degree of specialization of *T. moorii* and *X. boulengeri* may impede morphological changes in a trait (LPJ) that exhibits adaptive phenotypic plasticity even in later life stages in generalist cichlid species.

## CONFLICT OF INTEREST

None declared.

## AUTHOR CONTRIBUTIONS

**Lukas Widmer:** Formal analysis (lead); project administration (supporting); writing – original draft (equal). **Adrian Indermaur:** Conceptualization (equal); project administration (equal); supervision (equal); writing – review and editing (equal). **Bernd Egger:** Conceptualization (equal); investigation (equal); supervision (equal); writing – review and editing (equal). **Walter Salzburger:** Conceptualization (equal); funding acquisition (lead); investigation (equal); project administration (equal); supervision (equal); writing – original draft (equal).

## Supporting information

Figure S1‐S2

## Data Availability

Individual measurements for all specimens including microsatellite allele size and landmark coordinates for LPJ and body shape used in this study are available from the Dryad Digital Repository (https://doi.org/10.5061/dryad.9w0vt4bcf).
