# Supplementary material for: Where Am I? Niche constraints due to morphological specialization in two Tanganyikan cichlid fish species
Source: Ecol Evol. 2020 Aug 12;10(17):9410–8. doi: 10.1002/ece3.6629 (PMC7487241; doi:10.1002/ece3.6629)
Supplement: Supplementary file 1 — Figure S1‐S2 [file ECE3-10-9410-s001.pdf]

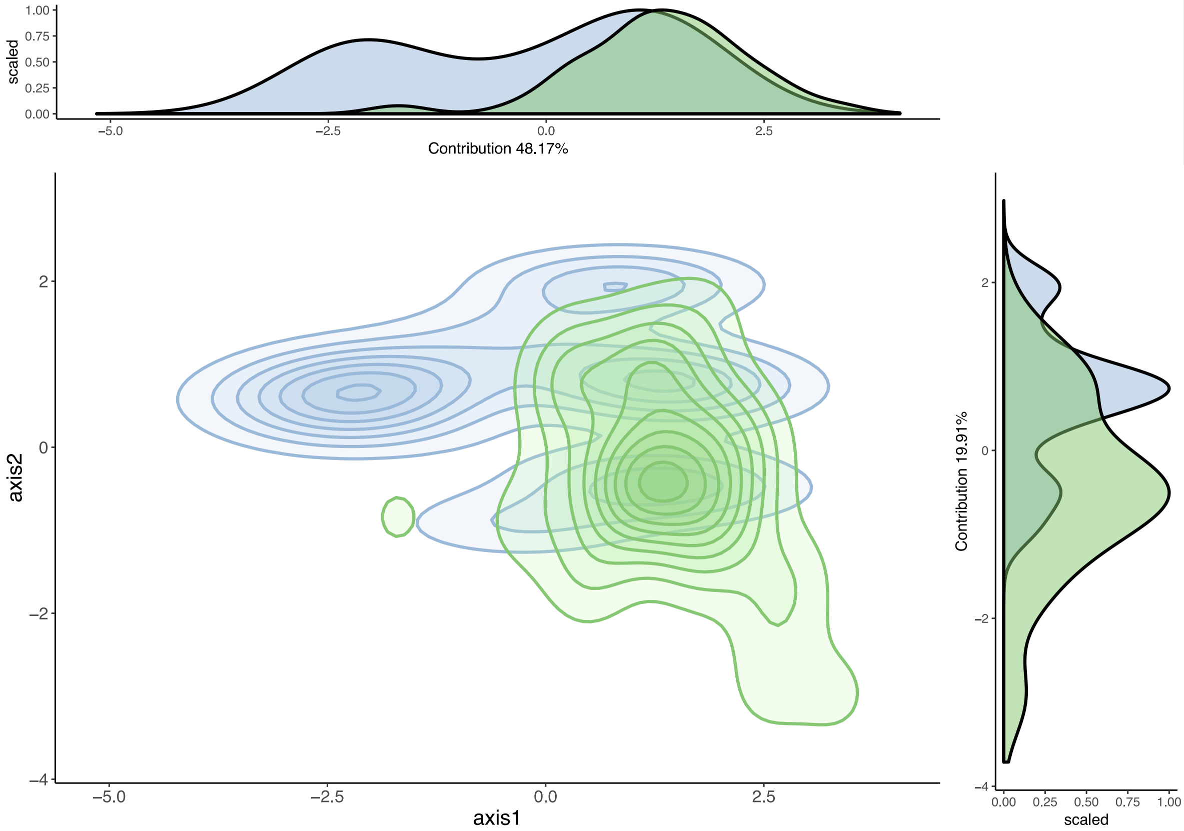

FIGURE S1 Niche overlap plot for occurrence density along PCA axes (axis1/axis2) based on *ecospat* analysis. Histograms on top shows densities for each species along PCA axis 1 and on the right along PCA axis 2 (PC2). Plot coloured for species *T. moorii* (green) and *X. boulengeri* (blue).

Cichlid census & niche Modelling

To confirm that *T. moorii* and *X. boulengeri* indeed occupy distinct ecological niches, cichlids occurrence data of a previous study were used to quantify and compare their ecological niches. Occurrence data for both species and the respective habitat parameters were extracted from a previous census study conducted at the same study site using Point-Combination Transects (PCT) (Widmer *et al.*, 2019). In brief, a PCT consists of five GoPro digital cameras in underwater housings (each taking one image per 10 s) placed on the benthos along a 40 m line, equally distanced from each other, at a target depth level. We used the cichlid census and environmental data extracted from these images and evaluated the differences in the ecological niche between *T. moorii* and *X. boulengeri*. To quantify habitat use, the environmental parameters of the cameras capturing *T. moorii*

(34 occurrences) and *X. boulengeri* (14 occurrences) were plotted against the environmental background, which was defined by the environmental parameters of the total number of 78 cameras. To evaluate niche overlap between *T. moorii* and *X. boulengeri* we calculated Schoener'*s D*, whereby a *D* of 0 equals no overlap and *D* of 1 equals complete overlap. Additionally, niche equivalency and similarity tests were performed with R package ecospat version 3.0 (replicates = 100) (Warren, Glor and Turelli, 2008).

Results

In the census data of Widmer et al. (2019) at the same study sites, *T. moorii* was found in water depths of 0.5 m to 14.7 m on predominantly rocky habitat (rock cover 71.4 – 100%), while *X. boulengeri* was found at slightly deeper depth levels (5 - 20.9 m) and roamed all substrate types (rock cover 0 – 100%) with a weighted $mean_{rock\ cover}$ = 47.6%. On six occasions both species were recorded on the same camera. Niche overlap for the two species was intermediate with Schoener's *D = 0.38* (Fig 2B). The test for niche equivalency revealed that the niches were not equal (p = 0.96), as was reciprocal testing for niche similarity for each species (1>2, p = 0.24; 2>1, p = 0.23). The ecological niches occupied by the two species used in this study have been described as rather distinct, whereby *T. moorii* is specialised to feed on aufwuchs on rocks and *X. boulengeri* to filter sand for macroinvertebrates (Konings, 2015). We can confirm these observations and reject the hypothesis of niche equivalency and similarity based on ecological niche model comparison on the basis of census data collected at the same study site.

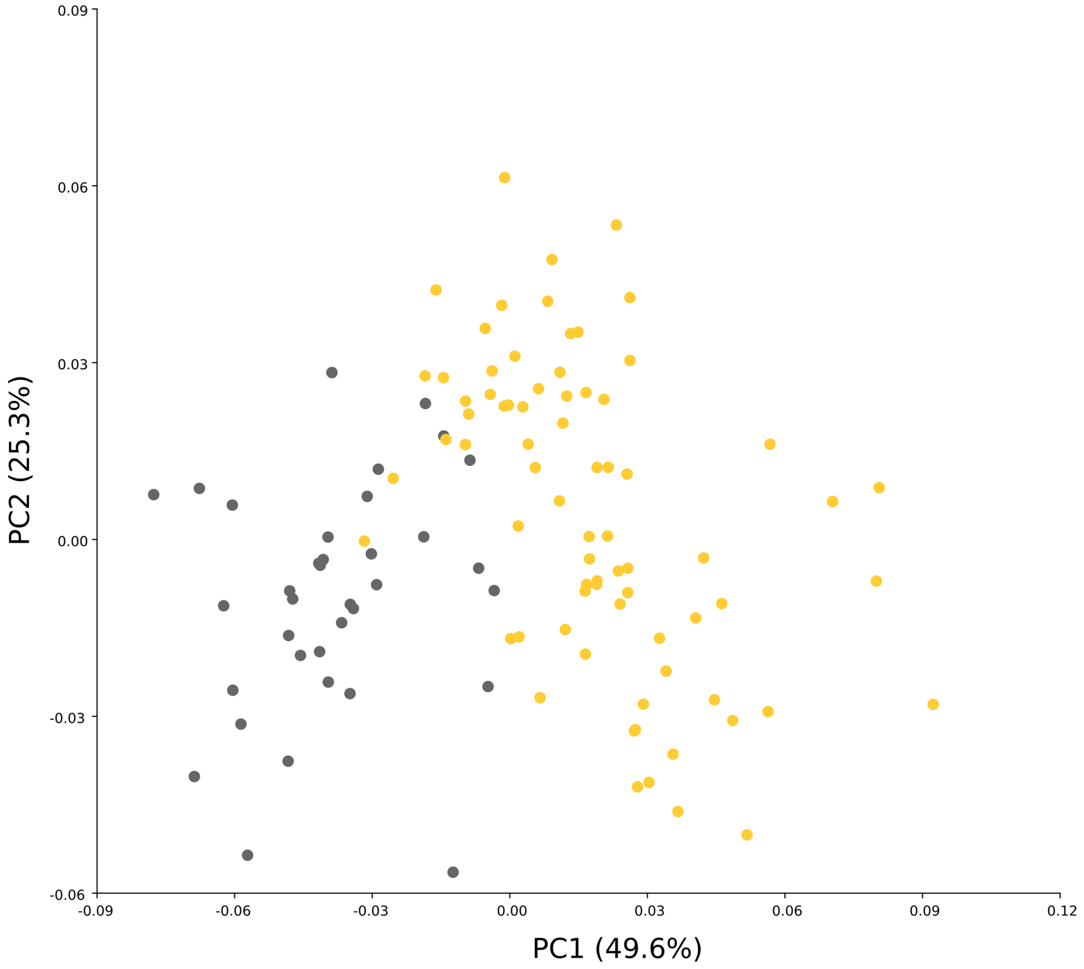

FIGURE S2 Principal component analysis based on 13 landmarks placed on LPJ. The entire data set of 111 specimens (1 outlier removed) of *T. moorii (black)* and *X. boulengeri (yellow)* (PC1 = 49.7 %, PC2 = 25.3 %)
